# Application and prospect of trabecular bone score in differentiated thyroid cancer patients receiving thyrotropin suppression therapy

**DOI:** 10.3389/fendo.2022.1004962

**Published:** 2022-10-12

**Authors:** Bingyu Ran, Feng Wei, Jian Gong, Hao Xu

**Affiliations:** Department of Nuclear Medicine, the First Affiliated Hospital of Jinan University, Guangzhou, China

**Keywords:** trabecular bone score, differentiated thyroid cancer, thyroid-stimulating hormone suppression therapy, fracture prediction, bone microarchitecture

## Abstract

Thyroid-stimulating hormone (TSH) suppression therapy is one of the common treatments for most patients with differentiated thyroid cancer (DTC). Unfortunately, its detrimental effects on bone health are receiving increasing attention. It may increase the risk of osteoporosis and osteoporotic fractures. The trabecular bone score (TBS) is a relatively new gray-scale texture measurement parameter that reflects bone microarchitecture and bone strength and has been shown to independently predict fracture risk. We reviewed for the first time the scientific literature on the use of TBS in DTC patients on TSH suppression therapy and aim to analyze and compare the utility of TBS with bone mass strength (BMD) in the management of skeletal health and prediction of fracture risk. We screened a total of seven relevant publications, four of which were for postmenopausal female patients and three for all female patients. Overall, postmenopausal female patients with DTC had lower TBS and a significant reduction in TBS after receiving TSH suppression therapy, but their BMD did not appear to change significantly. In addition, TBS was also found to be an independent predictor of osteoporotic fracture risk in postmenopausal women with DTC receiving TSH suppression therapy. However, due to limitations in the number of studies and study populations, this evidence is not sufficient to fully demonstrate the adverse effects of TSH suppression therapy on patients’ TBS or BMD and the efficacy of TBS, and subsequent larger and more case-cohort studies are needed to further investigate the relationship and application of TBS to TSH suppression therapy in terms of skeletal health impairment and fracture risk in DTC patients.

## Introduction

Thyroid cancer (TC) originates in the thyroid tissue, and DTC is the most common form of TC and also one of the most prevalent endocrine malignancies. In recent decades, DTC, mainly papillary carcinoma, has become one of the fastest-growing human cancers worldwide (1). Currently, the accepted treatment options for patients with DTC include initial thyroidectomy (total thyroidectomy or near-complete thyroidectomy and hemithyroidectomy or lobectomy with isthmus resection) (2), followed by radioiodine therapy (RAI), and long-term TSH suppression (3, 4). TSH suppression therapy is currently used mainly in patients with intermediate or high-risk TC or in patients with active disease (5). This approach has reduced the recurrence rate and cancer-related mortality in patients with DTC (4). However, some studies have shown that subclinical hyperthyroidism induced by TSH suppression therapy may have some adverse effects on patients, especially in the skeleton, leading to osteoporosis and an increased risk of osteoporotic fractures (6–10). Despite its high morbidity, DTC possesses a good prognosis with a high 10-year survival rate of more than 90% (1, 3, 11). Therefore, the clinical impact of long-term TSH suppression therapy on the skeleton is essential and is one of the most important factors affecting the prognosis of patients’ quality of life level.

Bone mineral density (BMD) measured by dual-energy x-ray absorptiometry (DXA) is the gold standard for the diagnosis of osteoporosis and the most widely used technique in treatment management (12–14). However, studies have shown that BMD has limitations in predicting osteoporotic fractures. BMD is overestimated when patients have scoliosis, lumbar osteoarthritis, vascular and/or joint calcification (15) and cannot account for the large number of low traumatic fractures that also occur in patients with osteopenia or even normal range BMD (16–19). Measurement of BMD alone is not a good estimate of the true severity of the bone disease. TBS is a parameter based on texture analysis of anterior and posterior DXA images of the lumbar spine and is closely related to the number of trabecular bones, and trabecular bone separation and connectivity, and other 3D bone structure parameters (20, 21). It can be used as an index for bone microarchitecture assessment and has the ability to independently predict the risk of low trauma fractures (22–25).

The use of TBS in the assessment of fragility fracture risk in patients with some secondary osteoporosis has been extensively validated, including diabetes (26–28), primary hyperparathyroidism (29), and rheumatoid arthritis (30), and chronic kidney disease (31, 32). Studies have shown that patients with DTC treated with long-term TSH suppression have lower TBS (33, 34). More importantly, they also found that TBS significantly predicted the relative fracture risk in patients with TSH suppression (35). TBS is likely to be a more sensitive variable as a way to assess bone health after TSH suppression therapy in patients with DTC.

This review is the first to review the literature on TBS and DTC to investigate the ability of TBS to assess bone health and predict fracture risk in DTC patients receiving TSH suppression therapy, to provide an early indicator of fracture risk for these patients, and to improve their clinical management options, thereby improving their quality of life.

## Method

PubMed was searched for English language papers using both free text and MESH terms (trabecular bone score, thyroid neoplasms, and thyrotropin). This identified 7 relevant papers that were examined in detail based on clinical relevance, leading to the following narrative summary.

## Thyroid hormones and bones

Thyroid hormones are tyrosine iodides synthesized by the thyroid follicular epithelium. The main ones are tetraiodothyronine (thyroxine, T4) and triiodothyronine (T3), in addition to small amounts of inverse-triiodothyronine (rT3). T4 has a longer half-life than T3 and needs to be converted to the active T3 (three to four times more potent than T4) within cells by deiodinases to function in skeletal development, linear growth, and adult bone turnover and maintenance of bone mass (36).

T3 binds to thyroid hormone receptor alpha (TRα), the main receptor expressed in bone, and stimulates osteoblasts directly or indirectly through a variety of growth factors and cytokines (37–39). Thyroid hormone receptors are also expressed in osteoclasts, and their interaction with T3 leads to an increase in the number and activity of osteoblasts, resulting in enhanced bone resorption. However, it is unclear whether the stimulation of osteoclast bone resorption by T3 is caused by direct action on osteoclasts or is a secondary result mediated by the main action of osteoblast spectrum cells (40, 41). Because RANKL expressed by T3-induced osteoblasts is the key osteoclast factor (42, 43). Excessive thyroid hormone action on osteoblasts results in increased activity of osteoblasts and osteoclasts, increased bone conversion rate, and shortened remodeling time, but a dominant role of increased osteoclast activity and a dysregulation of the dynamic balance between bone resorption and bone formation, leading to bone loss.

Thyroid hormones also affect bone mineral metabolism and, together with parathyroid hormone (PTH) and 1, 25- (OH) _2_D_3_ regulate and maintain osteocytes’ activity, bone reconstruction and calcium metabolic homeostasis. With hyperthyroidism, bone conversion is accelerated and bone resorption exceeds bone formation (44). The activation of bone resorption leads to an increase in serum calcium (45), which inhibits the synthesis of PTH and 1, 25- (OH) _2_D_3_ and the release of calcium, resulting in abnormal serum calcium concentrations. At the same time, some patients also often have hyperphosphatemia due to the release of phosphorus from bone and cartilage.

## TSH and bones

TSH is a glycoprotein hormone synthesized and secreted by thyroid cells in the anterior pituitary gland that exerts its regulatory effects by acting on the thyrotropin receptor (TSHR) on the cell surface. Its main function is to promote thyroid follicular cell development, synthesis and secretion of thyroid hormones. The TSH receptor, although expressed mainly on the basolateral membrane of thyroid follicular cells, is expressed in both osteoblasts and osteoclasts (46). It has been shown to bind TSHR expressed on osteoclasts, inhibit osteoclast formation and survival and prevent bone resorption mainly by reducing local tumor necrosis factor-α production (47–50), and also stimulate osteoblasts to accelerate bone formation (51, 52), while directly affecting bone remodeling with a potential protective effect on bone. Several clinical studies have shown a positive correlation between serum TSH levels and BMD (53–56) and a negative correlation with bone turnover index (57, 58), indicating that TSH may be an important protective factor against osteoporosis. A growing number of epidemiological studies have demonstrated a strong relationship between low TSH levels and fracture risk (59–62). For example, Bauer et al. found a 4. 5-fold and 3. 2-fold increased risk of vertebral and nonvertebral fractures, respectively, when serum TSH levels were <0. 1 µUI/mL (62).

## TSH suppression therapy

The basic principle of TSH suppression therapy is that TC cells express TSHR and TSH is a growth factor of thyroid follicular cells. A lower-than-normal serum TSH concentration may inhibit the growth, proliferation and spread of TC cells (63). Most believe that TSH suppression is indicated in patients with DTC with a high risk of recurrence (64, 65) and is beneficial in patients with distant metastases (66, 67), but its value in low-risk patients has not been demonstrated (64). Currently, the American Thyroid Association (ATA) recommends that serum TSH levels should initially be maintained below 0. 1µUI/mL for patients classified as high risk after primary treatment, and 0. 5-2 µUI/mL for patients with better initial treatment (5). In addition, TSH suppression therapy may have potential adverse effects on bone metabolism, especially in elderly patients (68, 69). This is because, during TSH suppression therapy, patients are in a subclinical hyperthyroid state with serum thyroid hormone levels in the normal range and suppressed serum TSH levels (≤0. 4 µUI/mL, especially <0. 1 µUI/mL) (70). Their bone resorption is stimulated, which may lead to an increased risk of bone loss, deterioration of bone microarchitecture and fragility fractures.

Many studies (71–73) have shown an increased risk of osteoporosis and osteoporotic fractures such as hip and spinal fractures in DTC patients treated with TSH suppression. For example, in a large population-based cohort study, Shin DW et al. (73) matched 185, 956 DTC patients on levothyroxine with normal subjects on a 1:1 propensity score. They showed that compared with matched comparison groups, high dose (HR 1. 25;95% CI, 1. 07 to 1. 45; risk difference 0. 31 more fractures per 1000 person-year;95% CI, 0. 09 to 0. 53) and low dose (HR 1. 05;95% CI, 0. 87 to 1. 27) thyrotropin-treated patients after thyroidectomy had an increased risk of osteoporotic fracture. More interestingly, they found that as a whole, DTC patients did not have a significantly increased risk of osteoporotic fractures (HR 1. 03; 95%CI, 0. 94 to 1. 12). Therefore, when treating DTC patients with TSH suppression, it is necessary to weigh the benefits of inhibiting tumor recurrence and progression against the risk of subclinical hyperthyroidism and to optimize the selection and management of fracture risk.

## TBS

Bone trabecular scoring (TBS) is a new grayscale texture measurement parameter, which measures the local change rate of gray level in 2D projected images rather than the direct measurement of 3D skeleton microarchitecture (74). The utility of TBS to estimate trabecular microarchitecture from existing DXA images of the lumbar spine without additional radiation exposure and at a lower cost has received considerable attention. As a non-invasive bone parameter that has been shown to be independent of BMD, TBS is able to reflect bone microarchitecture and, together with BMD, better reflect bone strength, thus contributing to the diagnosis of osteoporosis. The tipping point for TBS was proposed by analogy with three BMD categories (normal bone mass, osteopenia, and osteoporosis): TBS ≥ 1.31 was considered normal; TBS between 1.23 and 1.31 was considered consistent with partially degenerated microarchitecture; and TBS ≤ 1.23 was defined as degenerated microarchitecture. The cut-offs were considered to apply to women and men from 40 and above (including some premenopausal women) with no differences between sexes (75, 76). However, a large population study would be required to determine the optimal ranges across age and sex. In addition, TBS has been shown to be an independent predictor of primary osteoporosis-related fractures (77–79), and in combination with BMD, can better assess the risk of osteoporotic fractures (24). Also, a large number of studies have demonstrated that TBS plays an important role in the risk prediction of various secondary osteoporotic fractures (27–32, 36). More importantly, some studies have found a relatively significant decrease in TBS in patients who had no significant change or even an increase in BMD (80–82). This suggests that there are limitations in diagnosing secondary osteoporosis or predicting fracture risk by BMD alone and that TBS may be a more accurate predictor of fracture risk than BMD. As an evaluation tool of bone microstructure, TBS has a promising future in the diagnosis and fracture prediction of secondary osteoporosis. TBS has also been used in the FRAX tool to enhance personalized osteoporosis management and predict spinal and hip fractures and major osteoporosis fractures, helping to optimize treatment decisions (83). In addition, there have been some studies on the correlation between TBS and active substances in bone metabolism, and the ability of TBS to serve as a good indicator of response to treatment effects is under investigation (84–86).

## Related studies

### TBS and TSH suppression therapy

Several recent studies have shown that TSH suppression therapy is associated with a decrease in TBS in postmenopausal women with DTC (33–35, 87, 88) (**Table 1**). In a retrospective cohort study of 410 postmenopausal DTC patients with long-term TSH suppression, with or without osteoporosis, the TBS of TSH-inhibited patients was significantly lower than that of non-TSH-inhibited patients in the fourth year of examination. In patients with osteoporosis, moreover, greatly increased levels of TBS and BMD were observed in the TSH suppression (-), but there was no significant change in the TSH suppression (+) group. TBS even decreased significantly in the fourth year of follow-up (35). This may be due to the use of anti-osteoporotic drugs during follow-up, but TSH suppression therapy seems to counteract the anti-osteoporotic effects of drugs, which is consistent with the results of Annalisa Panico et al. (91) It suggests that for patients with TSH suppression (+), more attention should be paid to evaluating the effect of drug treatment when administering osteoporosis prevention therapy (35). A prospective study also showed that long-term TSH suppression could lead to the deterioration of trabecula in postmenopausal patients with DTC, but there was no correlation in premenopausal patients (33). Another study stratified 145 DTC women with a mean follow-up of 12. 3 ± 6. 1 years by the duration of TSH suppression, and found that the level of TSH suppression may be a major factor leading to the deterioration of TBS and BMD, and that TBS declined significantly earlier than BMD. This suggests that TBS may provide a more sensitive assessment of bone health in these patients. In a case-control study performed on 36 postmenopausal DTC patients with long-term TSH suppression (cases) and healthy postmenopausal women (controls), matched for age and body mass index (BMI), TBS in the cases was significantly lower in both examinations compared to the controls, while there was no significant difference in BMD (70). Moon et al. reported the results of a retrospective cohort study on 273 postmenopausal DTC women who received TSH suppression therapy. They found that the duration of TSH suppression correlated negatively with lumbar TBS and had an independent relationship. After adjusting for age, body mass index and BMD, the lumbar TBS of patients with TSH suppression duration ≥ 5 years was still significantly lower than that of patients with a duration < 3 years (34). However, no correlation was found in other studies (33, 88–90). As the authors explained, the reason may be that during long-term TSH suppression therapy, the level of TSH suppression is adjusted according to the patient’s prognosis and risk of recurrence, and only the duration of TSH suppression is not affected by other parameters, so it is independently related to TBS. However, there are different results in a cross-sectional study of 63 DTC Brazilian women by B. É. A. Sousa et al. They found that there was no significant difference in average TBS and BMD between the TSH suppression (+) group (43 patients) and the TSH suppression (-) group (20 patients). In their study, menopausal status and body mass index were the most associated variables for TBS. The possible reason is that the TSH of the patients included in the TSH suppression (-) group was in the low-normal range (a median TSH of 0. 800 µUI/mL and a 75th percentile of 1. 208 µUI/mL). However, studies have demonstrated that women with low-normal TSH have an increased risk of osteoporosis, and TSH may play a role in bone preservation, especially in postmenopausal women (59) (92, 93). In conclusion, TBS exacerbates more significantly than BMD in postmenopausal DTC patients treated with TSH suppression.

**Table 1:** Characteristics of the cohorts included in the study.

Study	Type	N (n)	Cohort	Age (year)	Duration of TSS (year)	Serum TSH (µUI/mL)	Summary of results
De Mingo Dominguez, M.L., et al., 2018^a*^ (33)	P	145 (84)	Caucasian	Baseline PRM: 41.98±8.5 PM: 58.68±8.6 Final visit PRM:45.71 ± 3.89 PM: 65.94 ± 9.29	10 (20)	Baseline PRM: 0.20 ± 0.42 PM: 0.25 ± 0.50 Final visit PRM: 0.41 ± 0.57 PM: 1.00 ± 1.77	Premenopausal DTC patients had a normal TBS significantly higher than that found in postmenopausal, showing postmenopausal patients' deterioration of bone microarchitecture. But serum TSH levels during TSS were not correlated with TBS.
Moon, J.H., et al., 2016^a^ (34)	RC	273 (273)	Korean	58.8 ± 6.9	4.2±2.2	0.05 (0.17)	Duration of TSS was negatively correlated with lumbar spine TBS levels (r=−0.180, p= 0.003), but not with BMD. TBSs were significantly lower in patients whose duration of TSS was ≥5 years compared with those whose duration was <3 years.
Chung, C.W., et al., 2021^a^ (35)	RC	410 (410)	Korean	Non-osteoporosis TSS−: 56.6 ± 6.6 TSS+: 56.6 ± 6.3 Osteoporosis TSS−: 61.0 ± 8.0 TSS+: 60.0 ± 8.0	≥2	Non-osteoporosis TSS−: 2.07 ± 1.15 TSS+: 0.46 ± 0.32 Osteoporosis TSS−: 2.51 ± 1.43 TSS+: 0.32 ± 0.25	Regardless of patients with osteoporosis or without osteoporosis, TBS was significantly lower in the TSS+ group than that in the TSS− group at year 4.
Kim, E.H., et al., 2019 (70)	RC	130 (130)	Korean	TSS: 60.5±5.5 Healthy control: 60.8±5.5	4.66±1.52	TSS: 0.00 (0.254) Healthy control: 1.975 (0.230)	The TBS and BMD did not differ significantly between the initial and follow-up DXA images in both groups of TSH suppressive patients and controls. TSS was revealed as not a significant factor for the progressive deterioration of bone status during long-term follow-up.
Hawkins Carranza, F., et al., 2020^a^ (88)	P	145 (131)	Caucasian	Baseline: 51.48 ± 11.9 Final visit: 63.96 ± 10.65	12.3±6.1	Baseline: 0.23 ± 0.4 Final visit: 0.89 ± 0.1	TBS values were lower in patients both with a follow‐up duration of 5‐10 years and >10 years of follow‐up compared with baseline values. TBS was significantly reduced in patients with TSS <0.1µUI/mL, whereas only non-statistically significant reductions in TBS were seen in patients with lower levels of TSS.
Sousa BÉ, C.A., et al., 2021 (89)	CS	63 (31)	Brazilian	sTSH: 49±13.8 nTSH: 51.7±11.7	4.0 (4.5)	sTSH: 0.059 (0.085) nTSH: 0.800 (0.686)	The TBS mean values were not significantly different in the sTSH and nTSH groups.​ BMI and menopausal status were the only variables associated with TBS and BMD.
Kim, K., et al., 2018^#^ (90)	CS	81 (81)	Korean	58 (3)	4.98 (1.48)	0.000 (0.002)	TBS was significantly lower in patients with osteopenia and osteoporosis than in those with a normal BMD value. However, the duration of TSS was not correlated with TBS.

Data are expressed as mean ± standard deviation or median (interquartile range).

N (n), total number of the study (number of postmenopausal); TSS, TSH suppression; TSH, Thyroid-stimulating hormone; P, prospective study; PRM, premenopausal; PM, postmenopausal; DTC, differentiated thyroid cancer; TBS, trabecular bone score; RC, retrospective cohort; BMD, bone mass strength; DXA, dual-energy x-ray absorptiometry; CS, cross-sectional study; sTSH, The suppressive therapy group was comprised of women with a mean TSH lower than 0.3 µUI/mL and free thyroxine (FT4)within the reference range; nTSH, The non-suppressive group included women with mean TSH equal to or greater than 0.3 µUI/mL, and FT4 within the reference range; BMI, body mass index.

a TSH suppression therapy and TBS were correlated in the study cohort.

^*^Correlation between TSH suppression therapy and TBS was only found in postmenopausal women.

^#^The study has not conducted this analysis.

### TBS and fracture prediction

TBS can be used as an independent indicator for the analysis of the bone structure to assess fracture risk (22, 79, 94). Multiple studies have shown that TBS combined with BMD is significantly superior to its use alone in assessing the risk of vertebral fracture (75, 95, 96). Current research explorations have found promise to further improve fracture risk assessment by TBS combined with FRAX (76, 97–99).

Chung et al. applied the Cox proportional hazards model and found that TBS could significantly predict the relative fracture risk in non-osteoporotic TSH-suppressed (+) patients: For each SD increase in TBS, the relative fracture risk was significantly reduced (92. 4%; 95% CI, 86. 1% to 99. 2%; p<0. 05) (35). However, no such significant predictive results were found in BMD (p=0. 628) and TSH suppression (-) (p=0. 843). B. É. A. Sousa et al. concluded that when assessed with TBS-adjusted FRAX, the probability of severe osteoporotic fractures was higher than that of FRAX without TBS (89). Meanwhile, some trials, although not directly examining the relationship between TBS and fracture incidence, showed a significant decrease in TBS at baseline in patients with TSH suppression (<0. 1 µUI/mL), suggesting that the use of TBS may serve as a synonym for fracture risk (88).

Assessment of TBS in DTC patients undergoing total thyroidectomy and receiving long-term TSH suppression may reveal trabecular bone deterioration, but a larger sample remains to be studied for the utility of TBS, TBS combined with BMD or FRAX in assessing bone fragility and potential fracture risk.

## Discussion

To our knowledge, this is the first literature review of the application of TBS in the assessment of skeletal health and prediction of fracture risk in DTC patients receiving TSH suppression therapy. We briefly describe the effects of thyroid hormones, TSH on human bone and the rationale for the application of TBS.

By reviewing relevant studies on the application of TBS in DTC patients, we analyzed in detail the relationship between TBS and DTC patients undergoing TSH suppression therapy and compared it with BMD, and also summarized the relationship between TBS and various related indicators. Currently, the effects of long-term TSH suppression therapy on the skeletal health aspects of DTC patients have not been definitively established, and there are different conclusions regarding studies assessing the risk of osteoporosis or fracture in DTC patients by TBS. When conducting research, we may need to consider the potential effects of weight changes on bone changes and image measurement parameters during TSH suppression. Initially, the image quality of DXA decreases with the increase of soft tissue thickness, and the higher the BMI, the lower the TBS (100, 101). Of the seven studies included in this review, four explicitly considered the effect of BMI (35, 88–90), well within the working range recommended for TBS (15 - 37 kg/m^2^) (102), while the remaining three did not. But the updated TBS algorithm (version 4) seems to overcome the residual negative correlation of TBS with body size and is suggested to be free from previously acknowledged technical limitations. Such newer versions of the TBS should be used in the future for more optimized research (103). Because the higher version is not yet widely used in clinical practice, BMI still needs to be taken into account when using the lower version of TBS. Meanwhile, the low level of serum vitamin D has been proved to be related to the decrease in bone mineral density, and the studies of Martineau P et al. also suggest that serum 25-OHD seems to be positively associated with TBS (87). In subsequent population studies, the bias caused by relevant biochemical indicators needs to be fully evaluated, and larger sample studies are needed to prove the relationship between long-term TSH suppression and changes in bone metabolic indicators.

In addition, due to the obvious ethnic differences in osteoporosis, the heterogeneity of study populations may have different results, and it is difficult to extend to other populations. Nevertheless, based on the current research results, TBS has a great potential for sensitive monitoring of bone health and even predicting the relative risk of fracture.

Based on relevant studies, TSH suppression therapy is highly likely to be a risk factor for bone health in DTC patients. Regular monitoring of bone health in these patients could be helpful in early screening for osteoporosis and maintaining life quality. A recent retrospective study in Korea evaluated the timing of repeated BMD testing in DTC patients with TSH suppression. They showed that for mild, moderate, and severe osteopenia, the estimated time interval for transition to osteoporosis was 85, 65, and 15 months, respectively, in female DTC patients over 50 years of age treated with TSH suppression (104). This appears to provide a useful basis for improving patient management. However, there are no such guidelines for TBS testing intervals to screen for osteoporosis in these patients. TBS was derived using the same dual-energy X-ray absorptiometry images as BMD for easy acquisition. Furthermore, there is no evidence to support that the use of TBS alone can guide treatment initiation. Therefore, we recommend that TBS be analyzed in conjunction with DXA follow-up testing to optimize the assessment of osteoporotic fracture risk and clinical risk factors.

The research on TBS and DTC that we have so far collected has focused on women, especially postmenopausal women. Physiologically, postmenopausal women with DTC suffer from the combined effects of estrogen deficiency and low TSH levels, resulting in increased bone resorption and an increased risk of osteoporosis and osteoporotic fractures. Menopausal status has also been shown to be a variable independently associated with TBS and BMD (89), having a significant impact on bone health. A recent meta-analysis of TSH suppression and BMD also showed that postmenopausal women receiving TSH suppression were at risk for bone loss, consistent with the results of most studies of TSH suppression therapy with TBS (105). This may explain why we are more inclined to investigate the bone health of postmenopausal women. Even so, we cannot ignore the attention to the bones of men and premenopausal women with DTC. Because TBS can evaluate bone microstructure texture, which may capture osteoporotic fracture risk not assessed by BMD, more studies are needed to prove the relationship between TSH suppression and TBS, though previous studies have shown that TSH suppression has no effect on BMD in these patients.

## Conclusions

Overall, despite the limited number of studies, the results suggest a possible association between TSH suppression therapy and the high risk of bone microstructural damage measured by TBS in postmenopausal women. Clearly, larger and better-designed studies reporting the effects of TSH suppression therapy on TBS are needed in the future to determine the impact of TSH suppression therapy on bone health and fracture risk in patients with thyroid cancer, so that the skeletal status of DTC patients can be assessed and monitored by BMD combined with TBS, which could help improve their clinical management and enhance the quality of life.

## Author contributions

All authors contributed to the study conception and design. Material preparation, data collection and analysis were performed by BR, JG and HX. The first draft of the manuscript was written by BR and FW, and all authors commented on previous versions of the manuscript. All authors read and approved the final manuscript.

## Acknowledgments

The authors acknowledge the support of the First Affiliated Hospital of Jinan University.

## Conflict of interest

The authors declare that the research was conducted in the absence of any commercial or financial relationships that could be construed as a potential conflict of interest.

## Publisher’s note

All claims expressed in this article are solely those of the authors and do not necessarily represent those of their affiliated organizations, or those of the publisher, the editors and the reviewers. Any product that may be evaluated in this article, or claim that may be made by its manufacturer, is not guaranteed or endorsed by the publisher.
